# Supplementary material for: CvkR is a MerR-type transcriptional repressor of class 2 type V-K CRISPR-associated transposase systems
Source: Nat Commun. 2023 Feb 18;14:924. doi: 10.1038/s41467-023-36542-9 (PMC9938897; doi:10.1038/s41467-023-36542-9)

CvkR is a MerR-type transcriptional repressor of class 2 type V-K CRISPR-associated transposase systems

Marcus Ziemann[1*], Viktoria Reimann[1*], Yajing Liang[2,3,4*], Yue Shi[2,3,4], Honglei Ma[2,3,4,5], Yuman Xie[2,5], Hui Li[2,5], Tao Zhu[2,3,4,5,#], Xuefeng Lu[2,3,4,5,6,#], Wolfgang R. Hess[1,#]

[1]University of Freiburg, Faculty of Biology, Institute of Biology III, Genetics and Experimental Bioinformatics, Schänzlestr. 1, D-79104, Germany;
[2]Qingdao Institute of Bioenergy and Bioprocess Technology (QIBEBT), Chinese Academy of Sciences, No.189 Songling Road, Qingdao 266101, China;
[3]Shandong Energy Institute, Qingdao 266101, China;
[4]Qingdao New Energy Shandong Laboratory, Qingdao 266101, China;
[5]University of Chinese Academy of Sciences, Beijing 100049, China;
[6]Laboratory for Marine Biology and Biotechnology, Qingdao National Laboratory for Marine Science and Technology, Qingdao 266237, China

Supplementary Material

Supplementary Figures

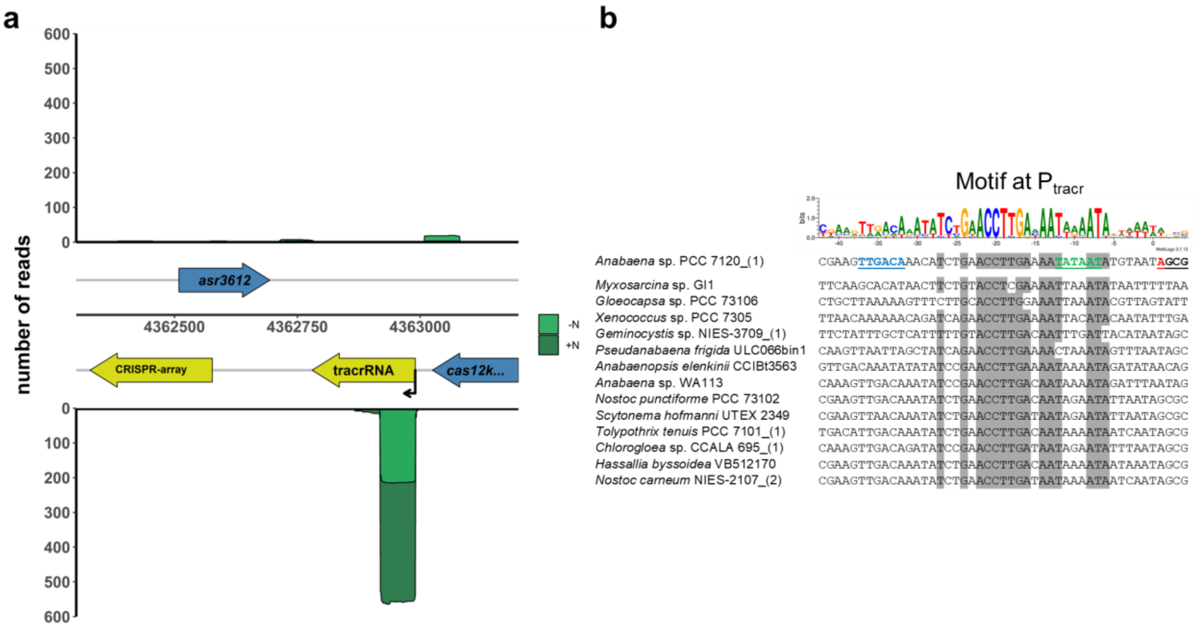

Supplementary Figure 1. The tracrRNA promoter (P_{tracr}). a Location of the tracrRNA TSS 35 nt downstream of *cas12k* in *Anabaena* 7120 according to the previous genome-wide mapping of TSS[1]. **b** Sequence conservation in the tracrRNA promoters of different CAST systems belonging to the same subgroup as the *Anabaena* 7120 CvkR according to the phylogenetic analysis (**Supplementary Fig. 3**). Positions with ≥92% conservation are shaded. Putative -10 and -35 elements of the promoter in the AnCAST system are colored green and blue, respectively, and the first tracrRNA nucleotides are underlined and in boldface letters (TSS in *Anabaena* 7120 is highlighted in red). One of the more closely related 15 systems according to **Supplementary Fig. 3** lacked a recognizable tracrRNA, therefore only 14 sequences were compared.

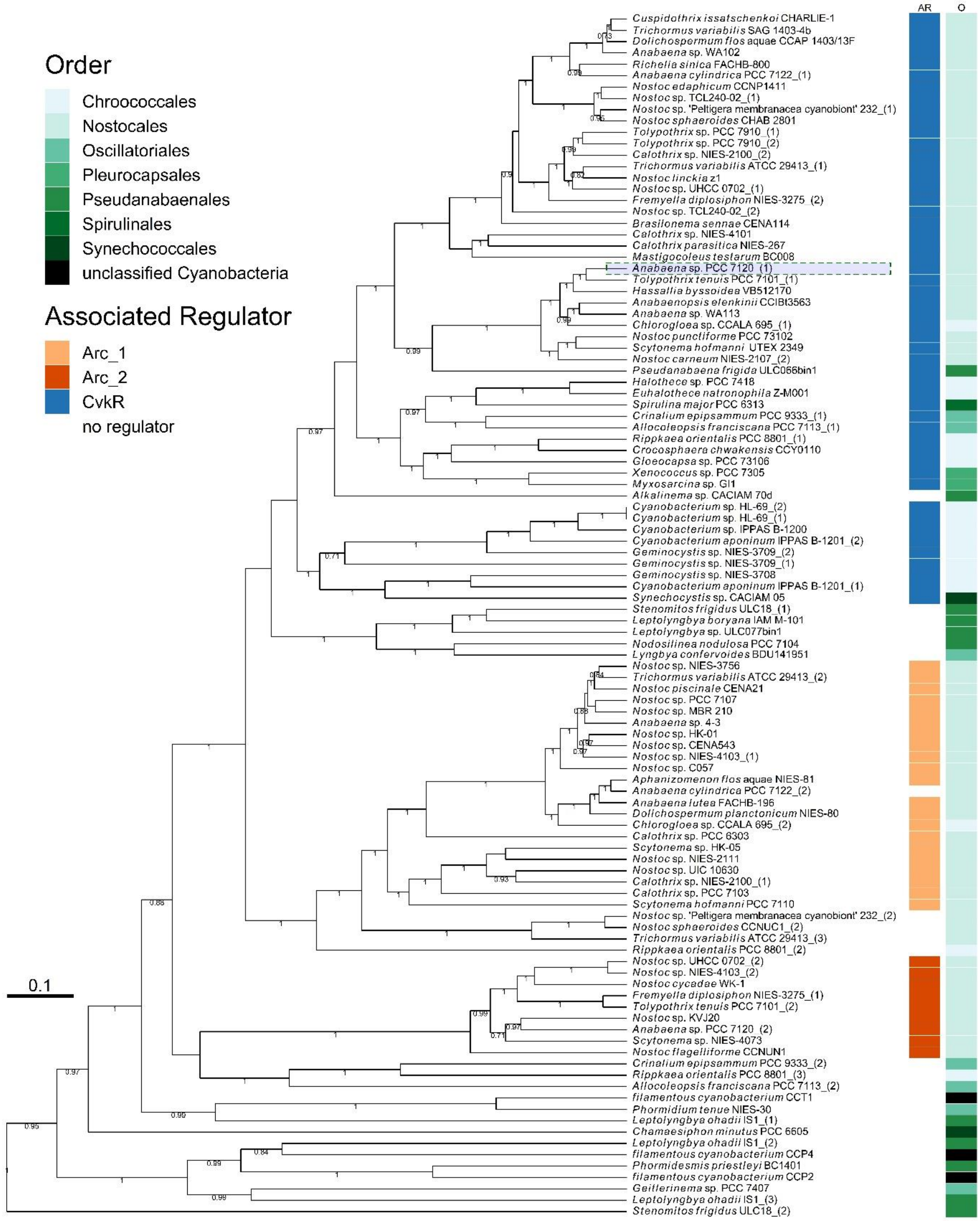

Supplementary Figure 2. Phylogenetic tree of Cas12k. The identified 106 complete Cas12k proteins were aligned using M-coffee[2,3] and analyzed by BEAST[4]. Eleven instances of degenerated Cas12k proteins were intentionally let out to avoid misinterpretation. The resulting tree is depicted with branches labeled with their respective posterior probability until a threshold of 0.7. For better recognition, the proteins were

labeled with their respective host organism (see **Supplementary Data 1**). The associated regulator type (AR) and taxonomic order (O) of the respective host organism are also depicted. The *Anabaena* 7120 Cas12k (All3613) (NCBI: BAB75312.1) is marked with a green dashed box. The multiple sequence alignments are available in **Supplementary Data 4**.

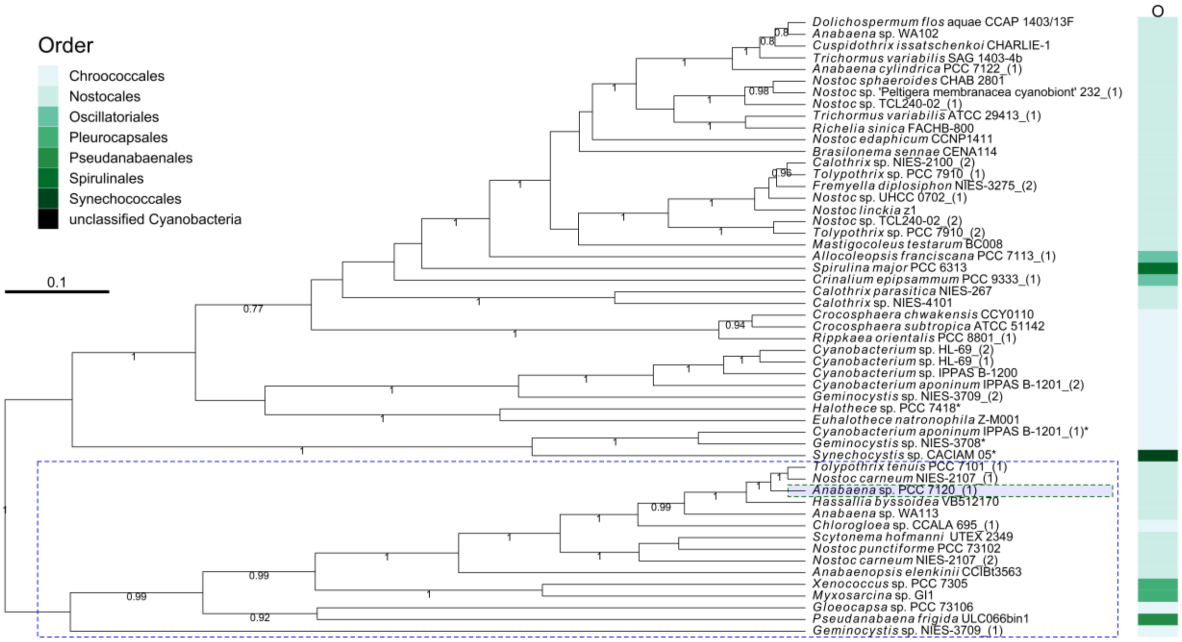

Supplementary Figure 3. Phylogenetic tree of CvkR. Phylogenetic tree of CvkR homologs. The proteins were aligned using M-coffee[2,3] and analyzed by BEAST[4]. The resulting tree is depicted with branches labeled with their respective posterior probability until a threshold of 0.7. For better recognition, the proteins were labeled with their respective host organism (see **Supplementary Data 1**). The taxonomic order (O) of the respective host organism are also depicted. *Anabaena* 7120 CvkR (Alr3614) (NCBI: BAB75313.1) is highlighted by a green dashed box and its most similar homologs by a blue-dashed box. Asterisks label four instances of CvkRs fused to an *hsdR* restriction enzyme domain. The multiple sequence alignments are available in **Supplementary Data 5**.

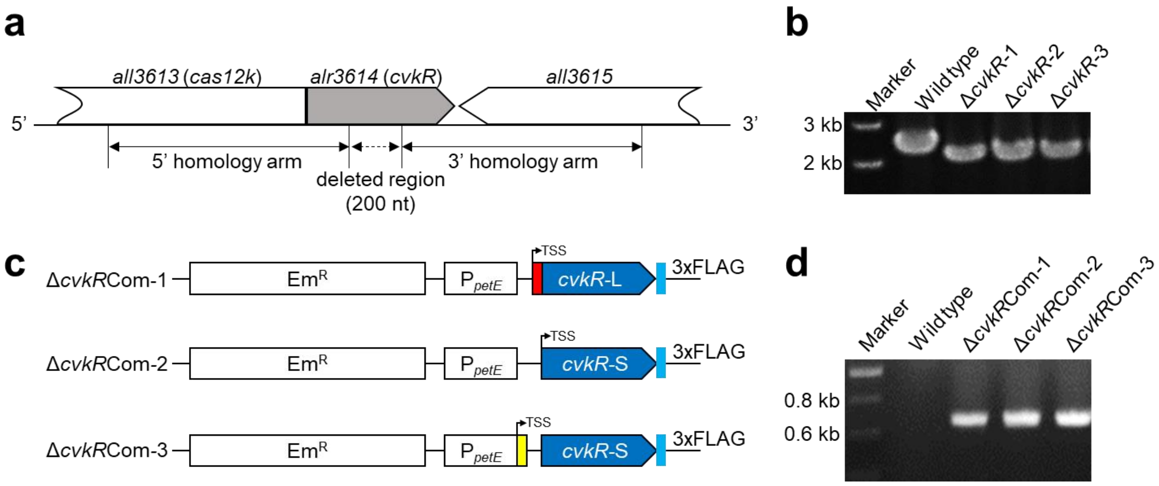

Supplementary Figure 4. Generation of *cvkR* deletion mutants and complementation of *ΔcvkR*. a Schematic drawing of the deleted regions and flanking regions of *cvkR*. **b** PCR assays showing successful segregation of deletion mutants in three independent replicates each. **c** Schematic drawing of the three cassettes for analyzing the leaderless expression of *cvkR*. The red bar indicates the extra 54-bp fragment at the 5'-end of *cvkR-L* compared to *cvkR-S*. The yellow bar indicates the 31 nt long 5'UTR of *petE*. **d** PCR assays showing successful construction of reporter strains for monitoring leaderless expression of *cvkR*. Please note that the result of one positive colony out of five (n=5) is shown for each reporter strain.

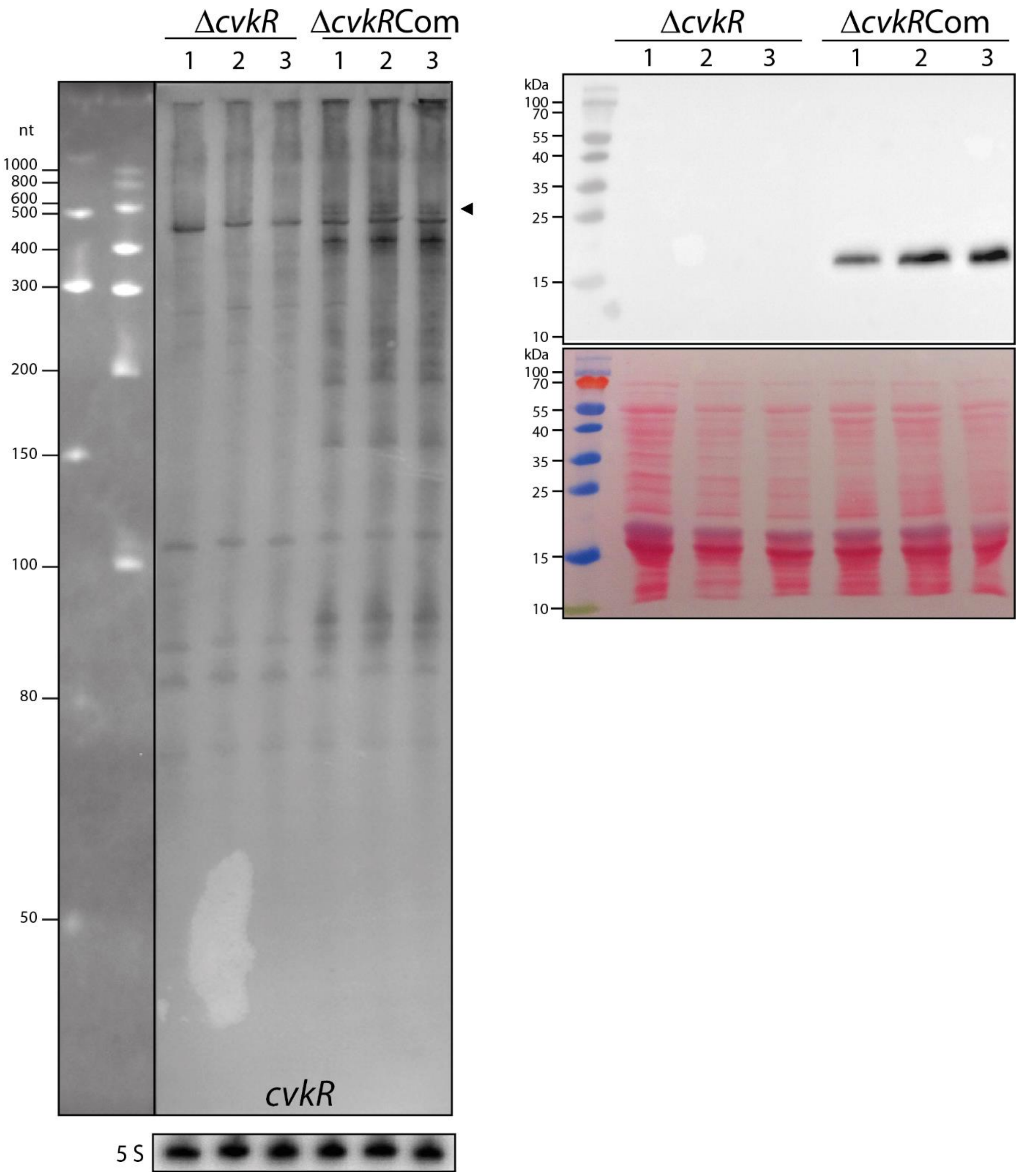

Supplementary Figure 5. Verification of absence or overexpression of CvkR in Δ*cvkR* or Δ*cvkR*Com, respectively. Left: Northern blot hybridization against *cvkR* in three independent replicates of Δ*cvkR* and Δ*cvkR*Com. The precise length of the *cvkR* mRNA is unknown because the transcript level in *Anabaena* 7120 WT is below the detection limit. The *cvkR* gene length is 453 bp. Taking the short-added sequence and

3'UTR into account, a correspondingly-sized additional signal in Δ*cvkR*Com represents the target *cvkR* band (labeled by triangle). Total RNA (20 µg) was loaded on a 10% PAA 8.3 M urea gel. The Low Range ssRNA Ladder (NEB) and the RiboRuler Low Range RNA Ladder (Thermo Fisher Scientific) were used as size markers. Right: Western blot against CvkR with an N-terminal 3xFLAG tag (upper panel) in three independent replicates of Δ*cvkR* and Δ*cvkR*Com. The stained membrane is shown in the lower panel. The calculated molecular mass for CvkR is 20.16 kDa. The prestained PageRuler (Thermo Fisher Scientific) was used as a size marker. Ten micrograms of total protein were loaded on a 15% SDS–PAA gel. Two separate experiments were performed, which showed consistent results.

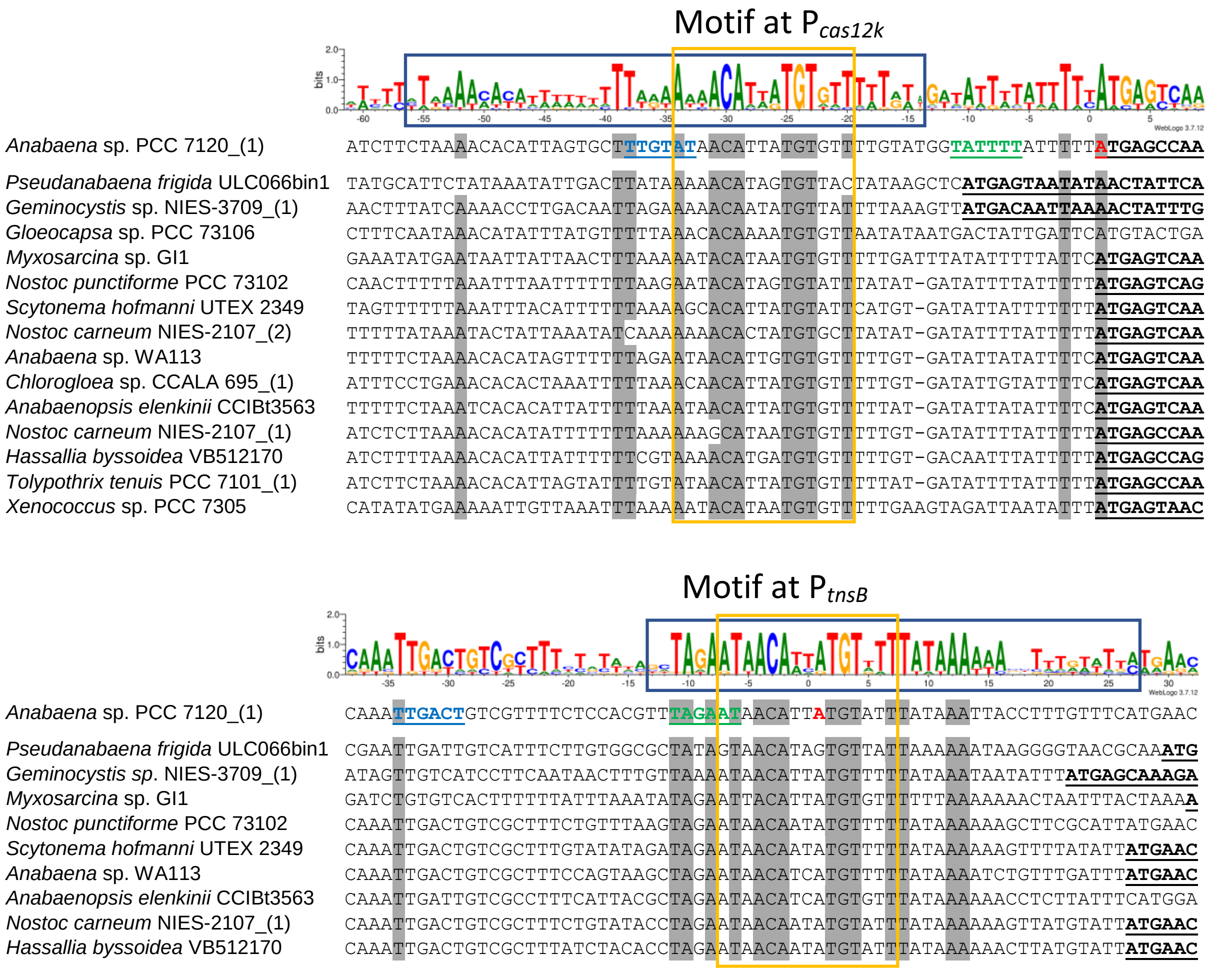

Supplementary Figure 6. Comparison of *cas12k* (top panel) and *tnsB* (lower panel) promoter homologs. The promoters of these genes belonging to the same CvkR subgroup as the AnCAST CvkR according to phylogenetic analysis (**Fig. 2b**) were compared to each other (≥92% conservation is shaded). Putative -10 and -35 elements of the genes in the AnCAST system are colored green and blue, respectively and the first codons of respective reading frames are underlined. The TSSs of the AnCAST genes are highlighted in red. Four of the observed CAST systems lack a *tnsB* gene and one *tnsB* was degraded, so the remaining 10 *tnsB* promoter sequences were aligned. The protected areas in the DNase I footprinting assays are blue boxed and the potential CvkR binding motif AnnACATnATGTnnT is yellow boxed.

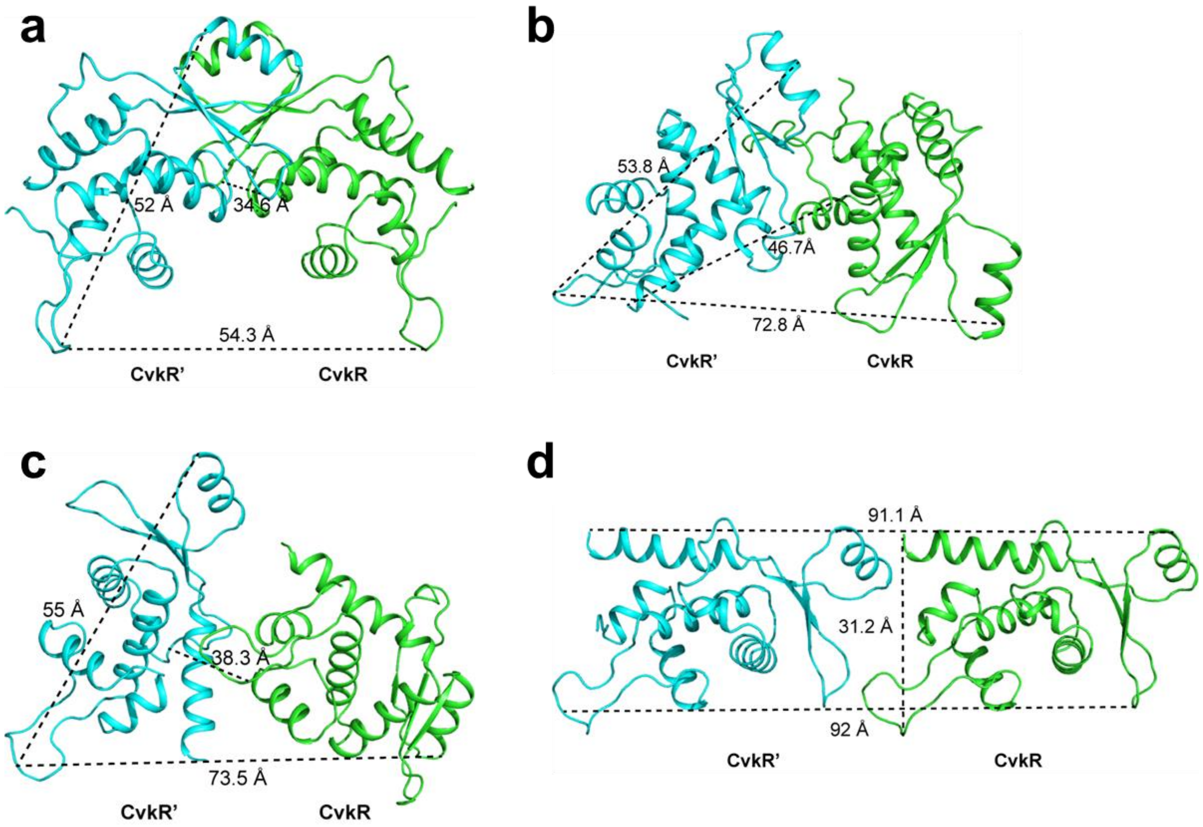

Supplementary Figure 7. Analysis of CvkR homodimer in structure. **a-d** four potential dimeric partners (CvkR', cyan) of CvkR (green) are found through different crystallographic symmetry operations. The resulting four homodimers have interface areas of 997.6 Å^2, 843.3 Å^2, 372.2 Å^2, and 276.6 Å^2, respectively, as calculated by webtool PDBePISA[5]. The sizes of CvkR-CvkR' homodimers are respectively further measured and labeled from several structural angles. The dimeric architecture, assembled by two vertically symmetric CvkR monomers shown in panel a, is similar to other reported MerR family members and is further approved by PISA assembly analysis and DLS assay.

.

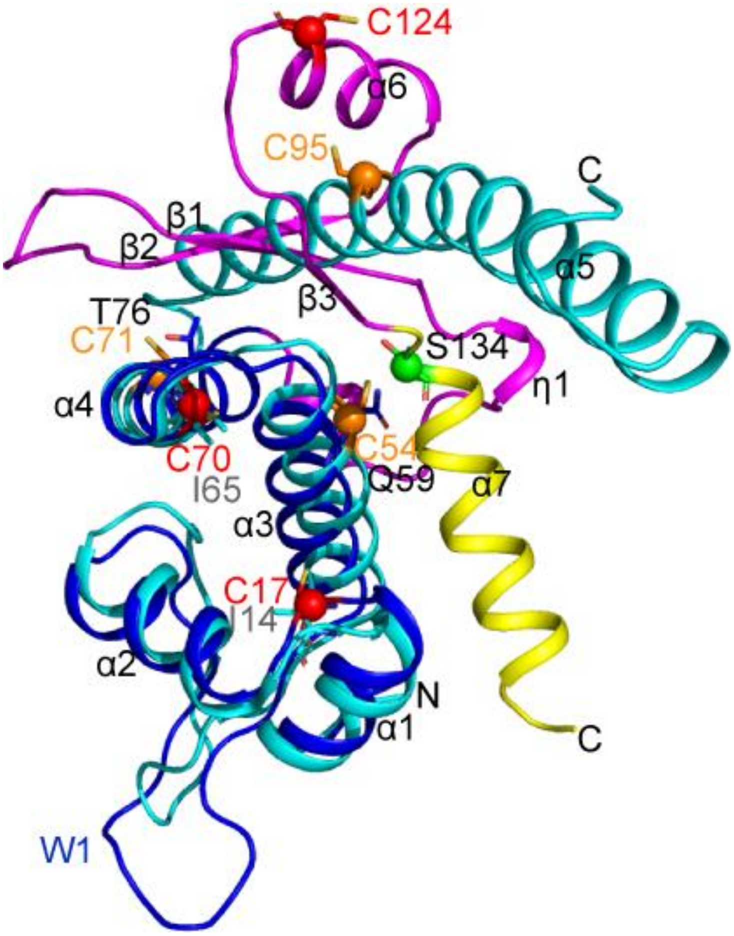

Supplementary Figure 8. Representation of cysteine sites in CvkR. The cysteine residues are represented in spheres and sticks and labeled with red in CvkR and with orange in HiNmlR (PDB code 5D8C, cyan). The Ser134 residue in CvkR is shown in green sphere and stick. Residues matching the positions of cysteines are shown in sticks and marked with black (CvkR) and gray (HiNmlR).

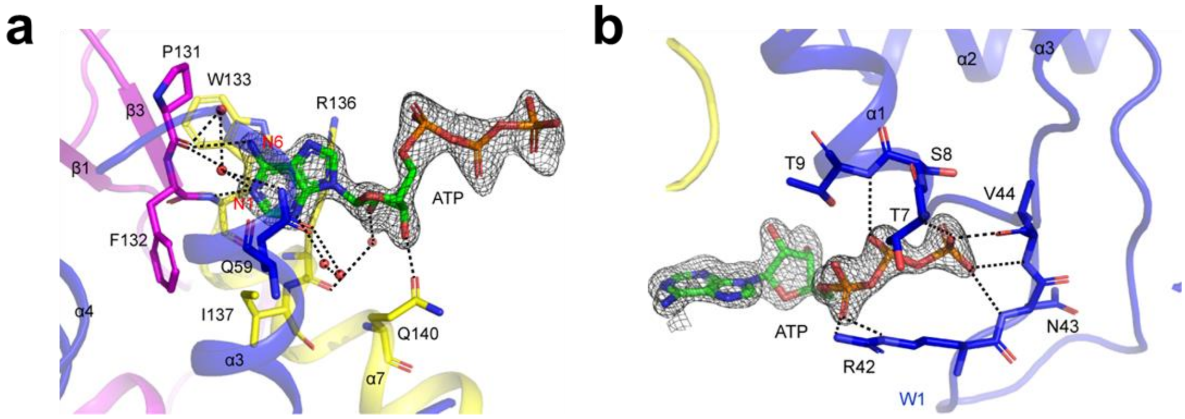

Supplementary Figure 9. ATP binding site of CvkR. a Close view of the ATP binding site in the CvkR monomer. **b** The ATP triphosphate group interacts with the wHTH domain of the adjacent CvkR monomer. The ATP ligand is represented in green stick and colored by the atom type. The N1 and N6 sites of ATP are labeled in red. The 2Fo-Fc density for the ATP is contoured in gray at 1.5 σ. The key residues involving in ATP binding are shown in sticks and labeled in black. Hydrogen bonds are indicated by dashed lines. The water molecules involved in hydrogen bonds are presented as red spheres.

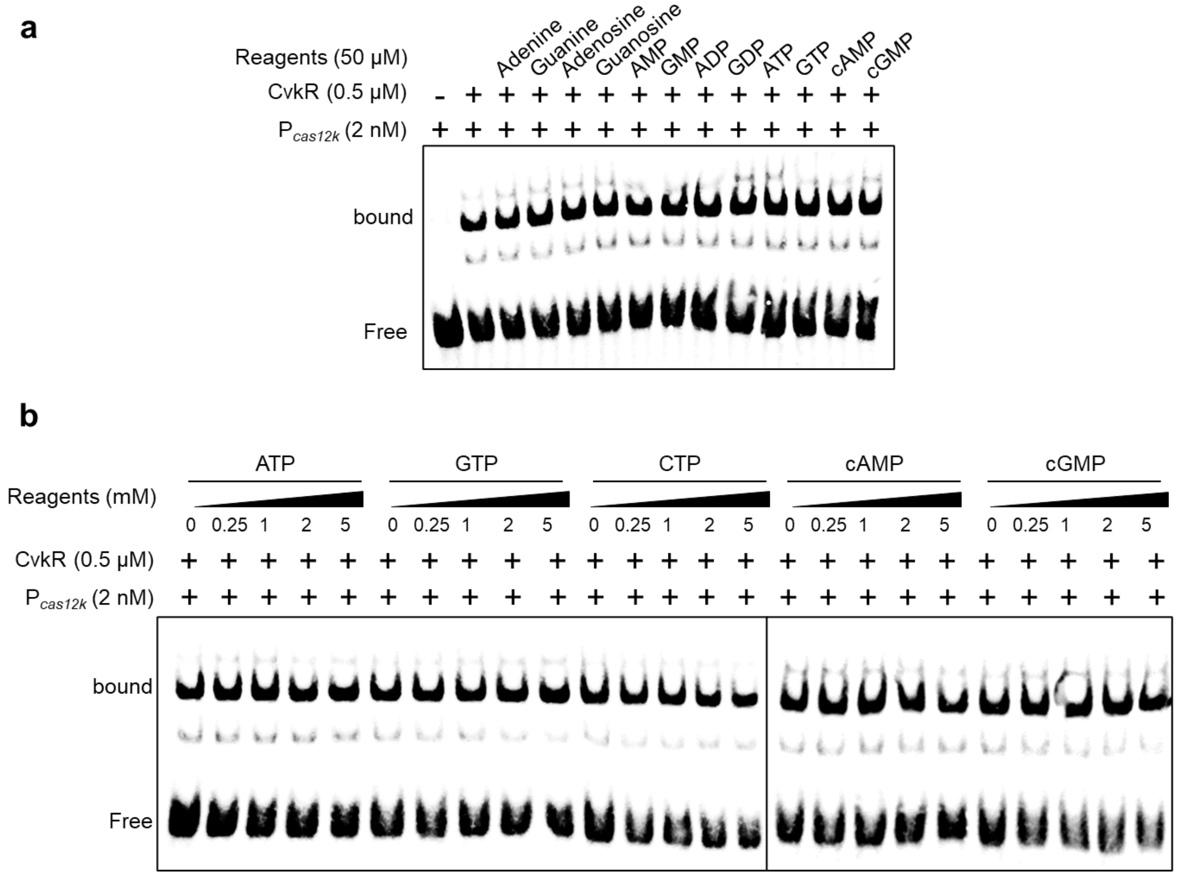

Supplementary Figure 10. EMSA assays showing the binding of CvkR to P_{cas12k} in the presence of multiple molecules with similar chemical structures to ATP. a EMSA showing the DNA binding ability of CvkR with the addition of adenine, adenosine, AMP, ADP, ATP, cAMP, guanine, guanosine, GMP, GDP, GTP and cGMP. **b** EMSA showing the DNA binding ability of CvkR with concentration gradients of ATP, GTP, CTP, cAMP, and cGMP. EMSA assays showing the binding of S134C to the *cas12k* promoter. The P_{cas12k} probe was used for the assay. The data are presentative from three independent experiments. The free probe and complexes of P_{cas12k} with CvkR are marked as "Free" and "Bound", respectively.

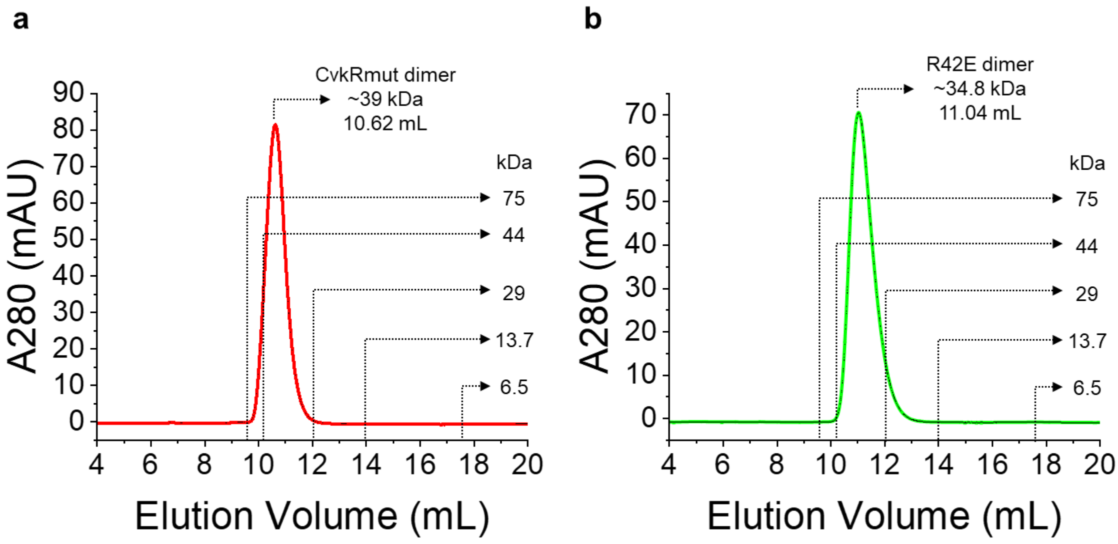

Supplementary Figure 11. Molecular weight estimates of CvkR mutant proteins by size exclusion chromatography (SEC) with Superdex 75 10/300 GL column (GE Healthcare). Calibration standards are indicated. **a** The elution volume of CvkRmut was 10.62 mL, corresponding to a mass of ~39 kDa. **b** The elution volume of R42E was 11.04 mL, corresponding to a mass of ~34.8 kDa. The estimated masses of both mutant proteins correspond to twice their calculated theoretical molecular weights.

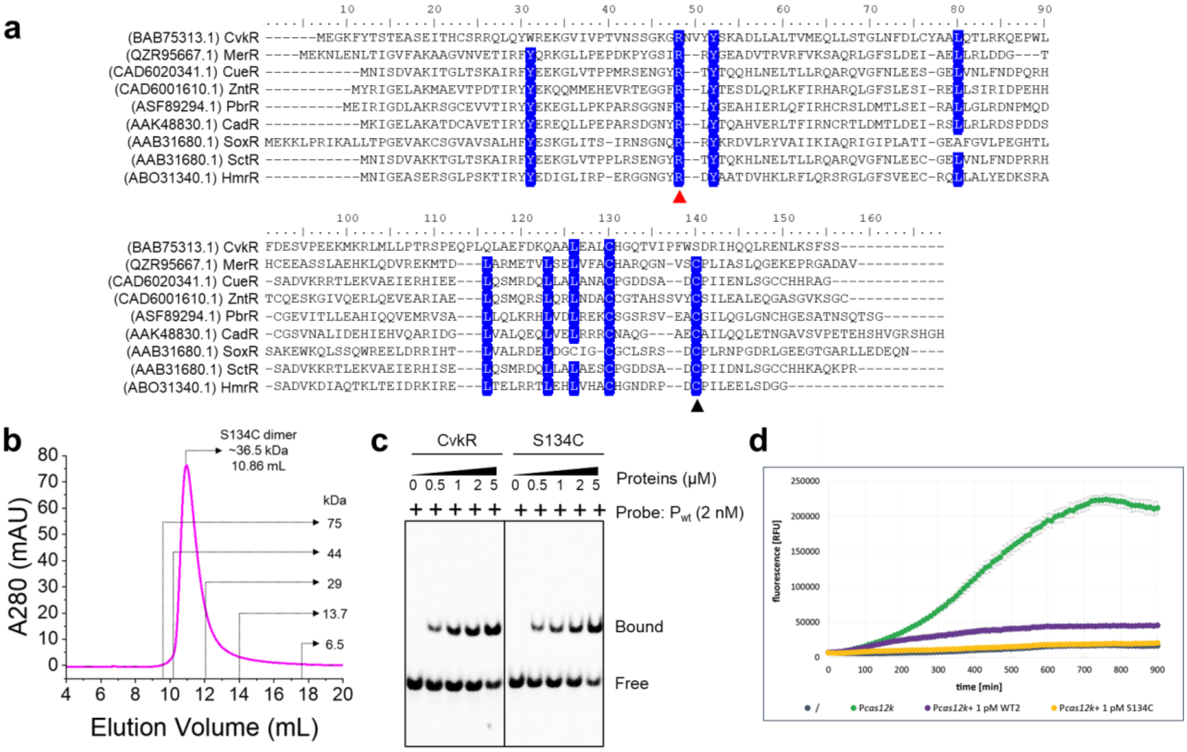

Supplementary Figure 12. Characterization of S134C mutant of CvkR. a Multiple sequence alignment of CvkR with selected MerR-type regulators. The NCBI accession numbers of the MerR-type regulators are given in brackets. The sequence alignment was performed using MAFFT[6], and visualized with BioEdit[7]. Threshold for shading is 80% identity. The black triangle indicates CvkR lacks a cysteine residue in the C-terminal sensor domain which is conserved in the selected members of the MerR-type regulators. The red triangle indicates one of the conserved arginine reported to interact with the phosphate backbone of promoter DNA[8,9]. **b** Molecular weight estimates of S134C mutant protein on SEC. Calibration standards are indicated. The elution volume of S134C was 10.86 mL, corresponding to a mass of ~36.5 kDa (twice its calculated molecular weight). **c** EMSA assays showing the binding of S134C to the *cas12k* promoter. The 33-nt P_{wt} probe was used for the assay. The data are presentative from three independent experiments. The free probe and complexes of P_{wt} with S134C are

marked as “Free” and “Bound”, respectively. **d** TXTL assay to test the regulatory capacity of S134C compared to CvkR WT2 for repressing deGFP fluorescence expressed from p70a under the control of P_{cas12k} (5 nM). Error bars show the standard deviation and are derived from 2 technical replicates. The experiments were repeated twice independently. The TXTL reactions were performed at 29°C overnight, and fluorescence was measured every 10 min in a Wallac 1420 Victor2 microplate reader. Error bars show the standard deviation derived from two technical replicates.

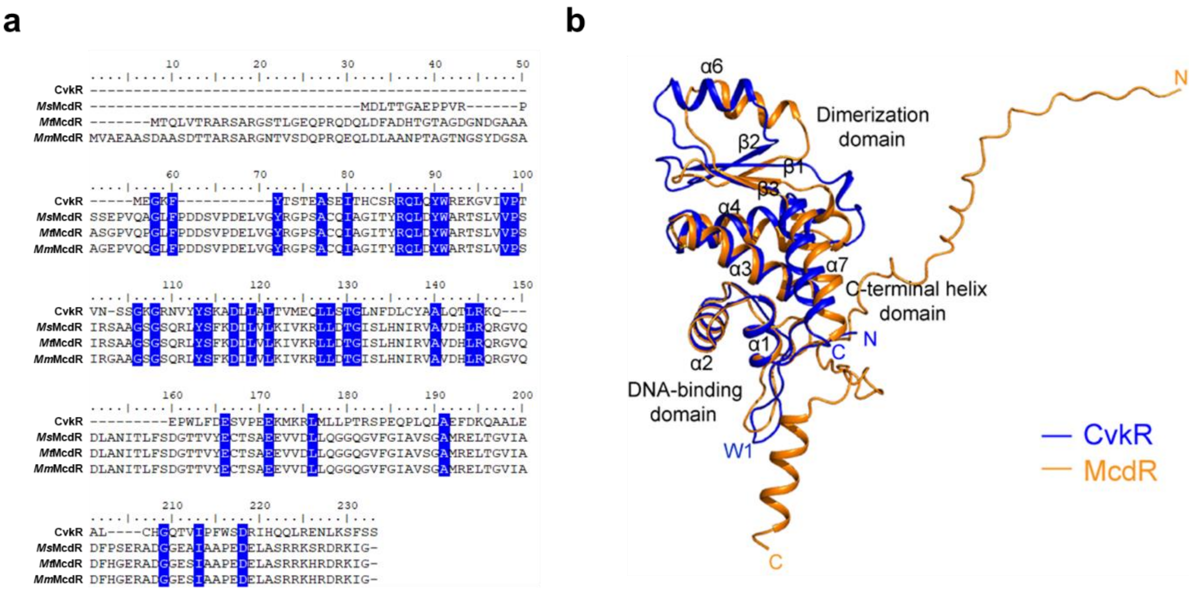

Supplementary Figure 13. Comparison of CvkR and McdR. **a** Multiple sequence alignment of CvkR and McdR. CvkR: Alr3614 of *Anabaena* sp. PCC 7120 (NCBI: BAB75313.1). MsMcdR: McdR of *Mycolicibacterium smegmatis* MC2 155 (NCBI: ABK74795.1). MtMcdR: McdR of *Mycobacterium tuberculosis* H37Ra (NCBI: ABQ73597.1). MmMcdR: McdR of *Mycobacterium marinum* M (NCBI: ACC41150.1). The sequences were aligned using MAFFT[2] and visualized with BioEdit[7]. **b** Superimposition of CvkR (blue, NCBI: BAB75313.1) onto AlphaFold2[10] predicted McdR (orange, NCBI: ABK74795.1) [https://alphafold.ebi.ac.uk/entry/A0QYF9]. The secondary structure elements and the DNA-binding, dimerization, and C-terminal helix domains of CvkR are labeled.

Supplementary Tables

Supplementary Table 1. Number of identified CAST associated regulator homologs and of CAST typical components in close vicinity.

	Total hits	near *cas12k*	near Left end	near CRISPR-array	near tracrRNA	near tRNA	no CAST-components
CvkR	157	130	93	61	85	100	11
Arc_1	32	25	22	19	24	25	5
Arc_2	21	14	15	10	10	14	2
total	210	169	130	90	119	139	18

Supplementary Table 2. Microarray analysis of Δ*cvkR* or Δ*cvkR*Com strains. Transcripts with significantly different accumulation levels in Δ*cvkR* compared to the complementation strain Δ*cvkR*Com. The microarray data was normalized using the R-implementation in package limma[11] and resulted p-values from t-tests were corrected using the Benjamini and Hochberg method to control the False Discovery Rate (FDR) of differential expression[12]. Threshold: $|\log_2| \geq 1$, p-value ≥0.01. Two replicates were used for the microarray experiment.

**Probe_Label**	**Gene/region_Function**	**location**	**log_2FC**	**adj. pvalue**
alr0738_igFwd	intergenic	chr	-2.413	1.41E-03
rtcB	RNA-splicing ligase	chr	-2.382	4.19E-03
cvkR	CvkR	chr	-2.203	4.77E-04
nTSS_52243_52243	tRNA L-array	delta	-1.987	4.77E-04
all8564	restriction endonuclease	delta	-1.743	1.41E-03
alr0739	uncharacterized conserved protein YdeI	chr	-1.616	5.37E-04
alr0740	slipin family protein	chr	-1.500	1.06E-04
asr0855	unknown	chr	-1.328	4.19E-03
all7121	cytochrome c domain-containing protein	alpha	1.075	1.41E-03
all0328_igFwd	intergenic	chr	1.109	3.81E-03
alr0786_igRev	intergenic	chr	1.268	3.28E-03
pecB	phycoerythrocyanin beta-chain	chr	1.300	4.19E-03
all0736_igRev	intergenic	chr	1.344	8.26E-03
alr1198_igFwd	intergenic	chr	1.387	1.12E-03
pecA	phycoerythrocyanin alpha-chain	chr	1.416	1.68E-03
pecC	phycoerythrocyanin-associated rod linker protein	chr	1.435	1.69E-04
tracrRNA	CAST tracrRNA	chr	1.493	4.33E-03
cas12k probe 1	CAST effector gene	chr	1.550	1.41E-03
all3391_igFwd	intergenic	chr	1.567	4.77E-04
tnsB	CAST transposase	chr	1.594	4.67E-04
cas12k probe 2	CAST effector gene	chr	1.870	9.16E-05

Supplementary Table 3. Oligonucleotide primers used in this work. Sequences belonging to the T7 promoter are underlined. Primers were purchased from IDT or Tsingke.

alr3614 deletion mutant (Δ*cvkR*) construction		
alr3614gRNA-1	AGATCATTAACAGTAATGGAGCAG	gRNA for *cvkR*
alr3614gRNA-2	AGACCTGCTCCATTACTGTTAATG	
alr3614KO-1	GGTCATTTTTTTGTCTAGCTTTAATGCGGTAGTTGGTACCGGTTAAGAGAATATCCTGCC	5' homology arm for *cvkR* knock out
alr3614KO-2	CAGGCGATCGCGTGGGTAATTACTATAACTCCTTTCTCTC	
alr3614KO-3	GAGAGAAAGGAGTTATAGTAATTACCCACGCGATCGCCTG	3' homology arm for *cvkR* knock out
alr3614KO-4	GCGCTGCCCGGATTACAGATCCTCTAGAGTCGACGGTACCAAAATCTTCGATGCAGATGA	
alr3614-3	GACGACTATCGCAGTAAACTTC	Genotype confirmation
alr3614-4	TTAGCTGCTTTACTACGACG	
alr3614 complementation strain (Δ*cvkR*Com) construction		
59M-F	GATTATAAAGATCATGATGGTGATTATAAAGATCATGATATTGATTATAAAGATGATGATGATAAATGAAAGGGTGGGCGCGCCGACCCAG	Amplification of pRL59EH-Cm/Em backbone with overlap to 3xFlag tag
59M-R1	GTTAATTTCACAGGCTTTAGGTCGACCTGCATCCCTTAAC	Amplification of pRL59EH-Cm/Em backbone with overlap to P_{petE}
P_{petE}-F	GTTAAGGGATGCAGGTCGACCTAAAGCCTGTGAAATTAAC	With overlap to pRL59EH-Cm/Em backbone
P_{petE}-R4	CACATTAGTGCTTTGTATAACATTTATTTCATTTTAAATAAAATCGACACC	$P_{petE\_no5'UTR}$-*cvkR-L*-3xFlag
P_{petE}-R5	GCTTGTGTAGAACTTTCCTTCCATTTATTTCATTTTAAATAAAATCGACACC	$P_{petE\_no5'UTR}$-*cvkR-S*-3xFlag
P_{petE}-R6	GTGTAGAACTTTCCTTCCATGGCGTTCTCCTAACCTGTAG	P_{petE}-*cvkR-S*-3xFlag
3614L-F	GGTGTCGATTTTATTTAAAATGAAATAAATGTTATACAAAGCACTAATGTG	$P_{petE\_no5'UTR}$-*cvkR-L*-3xFlag
3614S-F1	CGATTTTATTTAAAATGAAATAAATGGAAGGAAAGTTCTACACAAGC	$P_{petE\_no5'UTR}$-*cvkR-S*-3xFlag
3614S-F2	CTACAGGTTAGGAGAACGCCATGGAAGGAAAGTTCTACAC	P_{petE}-*cvkR-S*-3xFlag
3614-R	TCATTTATCATCATCATCTTTATAATCAATATCATGATCTTTATAATCACCATCATGATCTTTATAATCGCTACTAAAGCTTTTAAGATTC	Amplification of *cvkR*-3xFlag with overlap to pRL59EH-Cm/Em backbone
3614TesT-F	GCCGCCAGTTGCAGTATT	Genotype confirmation
3614TesT-R	CGGGCAAGTACGACATCA	
qRT-PCR		
alr3614 RT-F	GTGGCAAAGGTCGTAATGTT	qRT-PCR of *cvkR*
alr3614 RT-R	CGCTTCATCTTTTCTTCTGGG	
rnpB RT-F	CGTGAGGATAGTGCCACAGA	Internal standard
rnpB RT-R	CCAACCATAGTTCCTTCGGC	
Northern hybridizations		
CR_9_fwd	TTTGAATATTCAGAACTTTATATTGTGCGCGAT	as published[13], for mutant analysis

CR_9_rev	TAATACGACTCACTATAGGGGCAAGCTGATTTG GTAGAAGCTGTTAAT	for mutant analysis
all3613-Nblot1	GGAGTAAGCTTGGGGCTAGAA	for mutant analysis
all3613-Nblot2	TAATACGACTCACTATAGGGGCCTGCTTTATAGG TCTGTGC	
tracrRNA_Fw_T 7_1	TAATACGACTCACTATAGGGCTCTTTGGTGCGTC AAATCAAG	for mutant analysis
tracrRNA_Rev	CAGTTCATGCTGCTTGCAGC	
Alr3614_Nblot_f	GCACAGAAGCATCAGAAATTAC	for mutant analysis
Alr3614_Nblot_r	TAATACGACTCACTATAGGGAGACAGCAACTGC TCCATTAC	
5S_7120	TAGCAGCGTTTCACCTCTGAGTTCGG	as published[13]
**TXTL assay**		
pet28a_HisTEV _fwd	CTCGAGCACCACCACCAC	Amplification of pET-28a(+) with 6xHis tag and TEV site
pet28a_HisTEV _fwd	GGATCCCATATGCTGAAAATACAGG	
3614mut_fwd	ATTTTCAGCATATGGGATCCATGGAAGGAAAGTT CTACAC	Amplification of *cvkR*mut with overlaps to pET-28a(+)
3614mut_rev	TGGTGGTGGTGGTGCTCGAGTTAGCTACTAAAG CTTTTAAGATTC	
p70a-deGFP_fwd	GCTAGCAATAATTTTGTTTAACTTTAAGAAGGAG ATATACC	Amplification of p70a with deGFP and 5´UTR but without promoter
p70a-deGFP_rev	GCATGCCCAGCGGAACAG	
P3613_fwd	TGCTGTTCCGCTGGGCATGCAAGAAACATCCTA TAGAAGC	Amplification of the *all3613* promoter with overlaps to p70a
P3613_rev	TAAACAAAATTATTGCTAGCAAAAATAAAATACCA TACAAAACAC	
P3614_fwd	TGCTGTTCCGCTGGGCATGCAAAAATAAAATAC CATACAAAACAC	Amplification of the *alr3614* promoter with overlaps to p70a
P3614_rev	TAAACAAAATTATTGCTAGCAAGAAACATCCTAT AGAAGC	
P43_fwd	TGCTTTGTATAACATTATGTGTTTTGTATGGTATT TTATTTTTGCTAGCAATAATTTTGTTTAACTTTAA GAAGGAGATATACC	Amplification of p70a with deGFP under control of a shorter version of the *all3613* promoter
P43_rev	AAAAATAAAATACCATACAAAACACATAATGTTAT ACAAAGCAGCATGCCCAGCGGAACAG	
Ptracr_fwd	TGCTGTTCCGCTGGGCATGCCGCAGGATAAAGC AAAAG	Amplification of p70a with deGFP under control of the tracrRNA promoter
Ptracr_rev	TAAACAAAATTATTGCTAGCTATTACATATTATAT TTTCAAGGTTCAG	
PpsbAI_TXTL_f wd	TGCTGTTCCGCTGGGCATGCAAGGATTCCCAAA GATAGG	Amplification of p70a with deGFP under control of the *psbAI* promoter
PpsbAI_TXTL_r ev	TAAACAAAATTATTGCTAGCCTCATAAAATTTACA TGA	
P3630_TXTL_fw d	TGCTGTTCCGCTGGGCATGCGTCGCTTCAGTAA TTACAAAAAAG	Amplification of p70a with deGFP under control of the *all3630* promoter
P3630_TXTL_re v	TAAACAAAATTATTGCTAGCAATTTATAAATACAT AATGTTATTCTAAACG	
**Alr3614S protein heterologous expression**		
3614S-BamHI-F	CGGGATCCATGTTATACAAAGCACTAATGTG	Producing 3614S-pET-28a(+)-smt3
3614S-taa-XhoI-R	CCGCTCGAGTTAGCTACTAAAGCTTTTAAGATTC TC	
**Promoter fragments for TXTL assay**		

P_{cas12k}	AAGAAACATCCTATAGAAGCATCTTCTAAAACACA TTAGTGCTTTGTATAACATTATGTGTTTTGTATGGT ATTTTATTTTT	TXTL
P_{tracr}	CGCAGGATAAAGCAAAAGAATTAGCCCTCTATGC TTATAGTCTCCGCCTAGCTAGGCGAAGTTGACAA ACATCTGAACCTTGAAAATATAATATGTAATA	TXTL
P_{cvkR}	AAAAATAAAATACCATACAAAACACATAATGTTATA CAAAGCACTAATGTGTTTTAGAAGATGCTTCTATA GGATGTTTCTT	TXTL
P43	TGCTTTGTATAACATTATGTGTTTTGTATGGTATTT TATTTTT	TXTL
P_{psbAI}	CAAGGATTCCCAAAGATAGGGGGAATAATTAACA TTAAGAATTATTAATTCATGGGTTTTTAGTCTAGTA AATTTGCGTGAATTCATGTAAATTTTATGAG	TXTL
P_{tnsB}	GTCGCTTCAGTAATTACAAAAAAGGTTTTGTATAT TTTCATAATGACAAATTGACTGTCGTTTTCTCCAC GTTTAGAATAACATTATGTATTTATAAATT	TXTL
DNase I footprinting		
P3613-F(FAM)	AACTCCTTTCTCTCGCCAATAC	Producing FAM-labeled probes
P3613-R	GCTGCTGAAGCAGTTCGTT	
M13F(FAM)	CCCAGTCACGACGTTGTAAAACG	Producing FAM-labeled probes
M13R	AGCGGATAACAATTTCACACAGG	
CvkR protein mutant construction		
CvkRMut-F	GGTGGTCATATGGGATCCATGGAAGGAAAGTTCT ACAC	CvkRmut mutant
CvkRMut-R	CGTTTAGAGGCCCCAAGGGGTTATGCTAGTTATT GCTCAG	
pET28a-sumo-F	GAGCAATAACTAGCATAACCCCTTGGGGCCTCTA AACGGG	
pET28a-sumo-R	GTAGAACTTTCCTTCCATGGATCCCATATGACCA CCAA	
R42E-F	TTAACAGTAGTGGCAAAGGTGAAAATGTTTACT	R42E mutant
R42E-R	AGTAAACATTTTCACCTTTGCCACTACTGTTAA	
S134C-F	AAACTGTGATTCCTTTTTGGTGCGATCGCATTCA TCAACAA	S134C mutant
S134C-R	GCACCAAAAAGGAATCACAGTTTGTCCATGAC	

Supplementary Table 4. Data collection and refinement statistics.

	SeMet CvkR	Native CvkR
**Data collection**		
Space group	C121	C121
Cell dimensions		
a, *b*, *c* (Å)	78.813, 49.778, 41.017	78.579, 49.703, 40.52
α, β, γ (°)	90, 116.38, 90	90, 116.044, 90
Resolution (Å)	24.90-1.93 (2.03-1.93)	50.00-1.60 (1.66-1.60)
R_{sym} or R_{merge}	0.054(0.25)	0.054(0.441)
$I / \sigma I$	14.6 (3.8)	32.1 (2.93)
Completeness (%)	94 (84.4)	99.8 (99.2)
Redundancy	3.2 (3.1)	2.5 (2.6)
**Refinement**		
Resolution (Å)		1.6
No. reflections		18599
R_{work} / R_{free}		0.194/0.217
No. atoms		
Protein		1199
Ligand/ion		38
Water		92
B-factors		
Protein		45.39
Ligand/ion		45.50
Water		51.62
R.m.s. deviations		
Bond lengths (Å)		0.01
Bond angles (°)		1.29

Values in parentheses are for highest-resolution shell.

Supplementary References

1. Mitschke, J., Vioque, A., Haas, F., Hess, W. R. & Muro-Pastor, A. M. Dynamics of transcriptional start site selection during nitrogen stress-induced cell differentiation in *Anabaena* sp. PCC7120. *Proc. Natl. Acad. Sci.* **108**, 20130–20135 (2011).
2. Di Tommaso, P. *et al.* T-Coffee: a web server for the multiple sequence alignment of protein and RNA sequences using structural information and homology extension. *Nucleic Acids Res.* **39**, W13–W17 (2011).
3. Notredame, C., Higgins, D. G. & Heringa, J. T-coffee: a novel method for fast and accurate multiple sequence alignment. *J. Mol. Biol.* **302**, 205–217 (2000).
4. Suchard, M. A. *et al.* Bayesian phylogenetic and phylodynamic data integration using BEAST 1.10. *Virus Evol.* **4**, (2018).
5. Krissinel, E. & Henrick, K. Inference of macromolecular assemblies from crystalline state. *J. Mol. Biol.* **372**, 774–797 (2007).
6. Katoh, K. & Standley, D. M. MAFFT Multiple Sequence Alignment Software version 7: improvements in performance and usability. *Mol. Biol. Evol.* **30**, 772–780 (2013).
7. Hall, T. A. BioEdit: a user-friendly biological sequence alignment editor and analysis program for Windows 95/98/NT. *Nucleic Acids Symp. Ser.* **41**, 95–98 (1999).
8. Changela, A. *et al.* Molecular basis of metal-ion selectivity and zeptomolar sensitivity by CueR. *Science* **301**, 1383–1387 (2003).
9. Liu, X. *et al.* Selective cadmium regulation mediated by a cooperative binding mechanism in CadR. *Proc. Natl. Acad. Sci. U. S. A.* **116**, 20398–20403 (2019).
10. Jumper, J. *et al.* Highly accurate protein structure prediction with AlphaFold. *Nature* **596**, 583–589 (2021).
11. Ritchie, M. E. *et al.* limma powers differential expression analyses for RNA-sequencing and microarray studies. *Nucleic Acids Res.* **43**, e47 (2015).

12. Benjamini, Y. & Hochberg, Y. Controlling the false discovery rate: a practical and powerful approach to multiple testing. *J. R. Stat. Soc. Ser. B Methodol.* **57**, 289–300 (1995).

13. Hou, S. *et al.* CRISPR-Cas systems in multicellular cyanobacteria. *RNA Biol.* **16**, 518–529 (2019).

Uncropped scans of blots and gels in Supplementary Figures

Supplementary Figure 5.

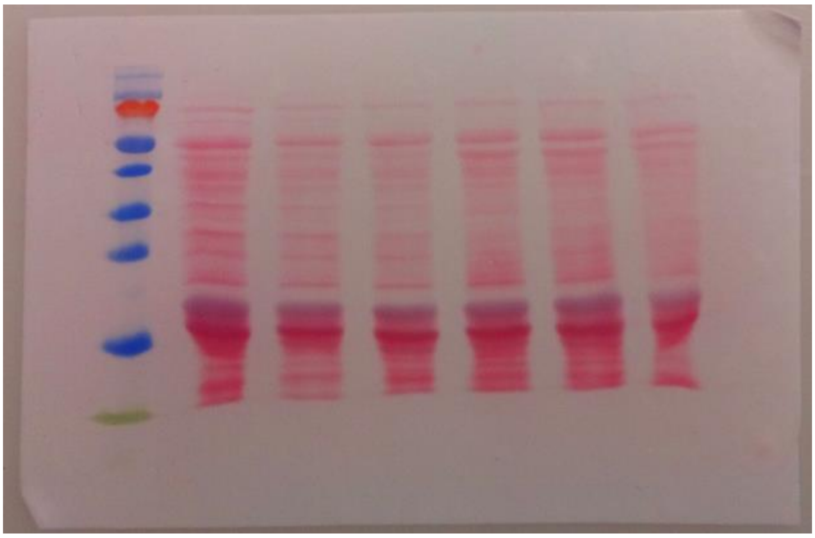

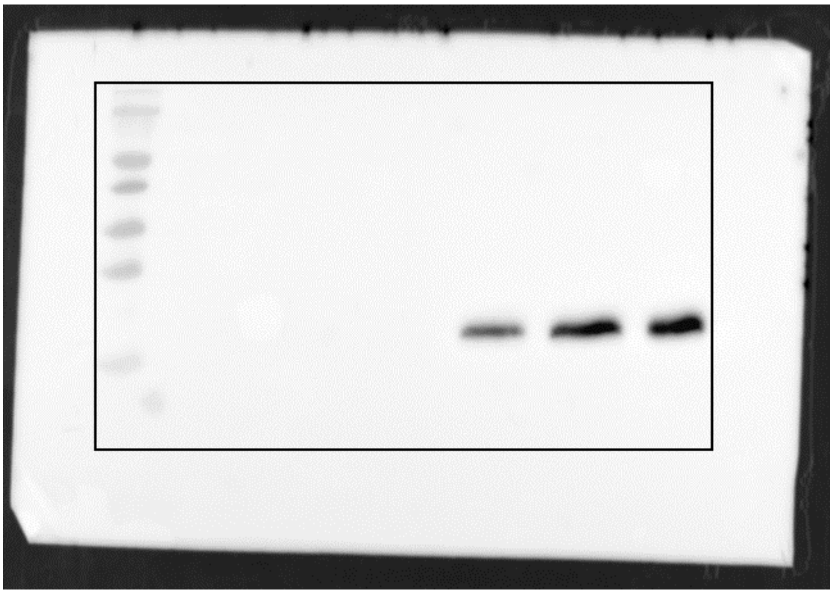

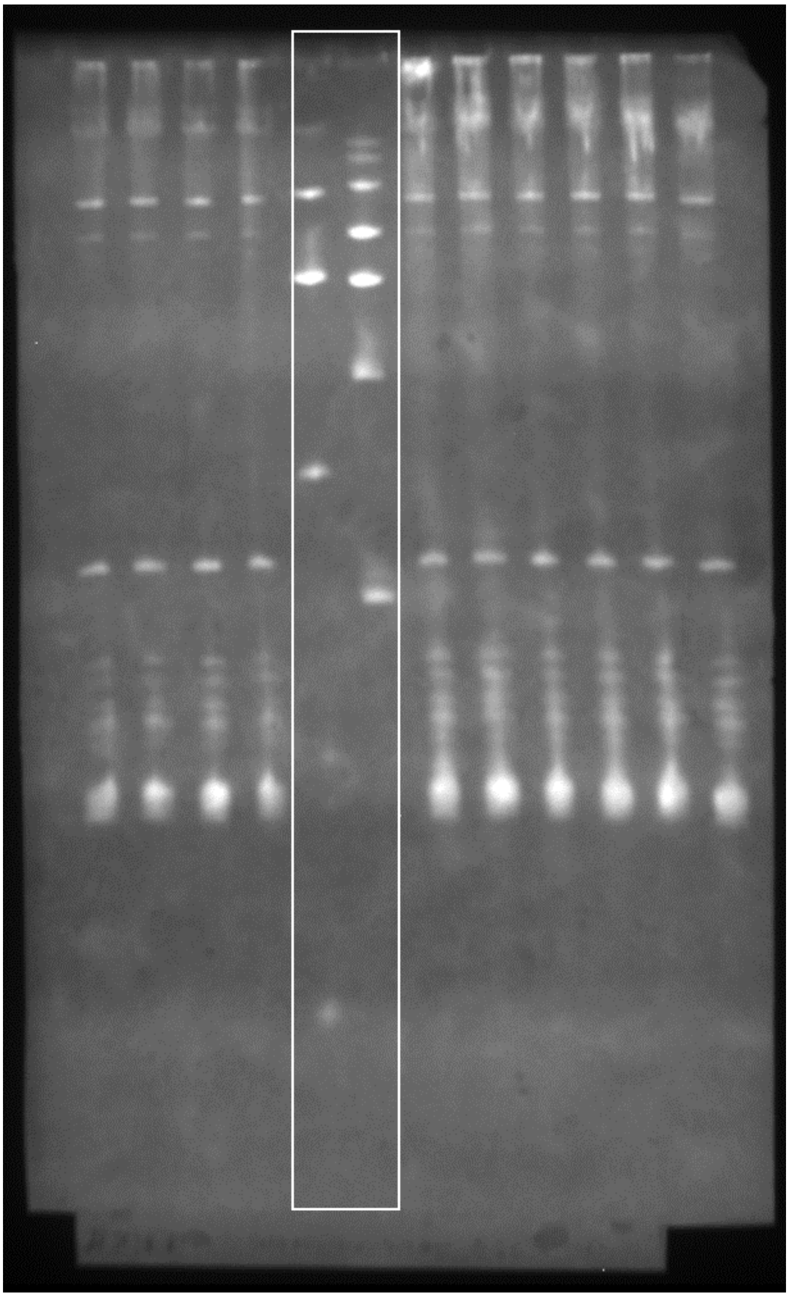

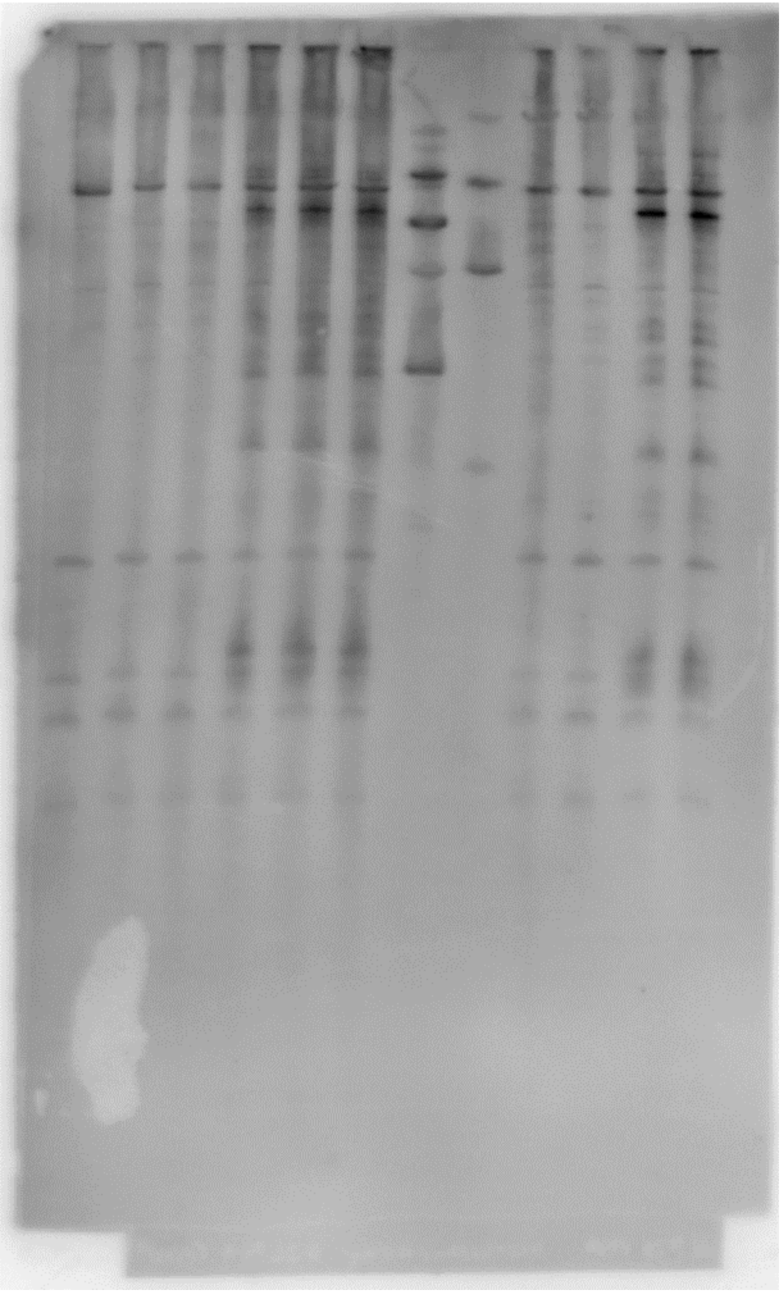

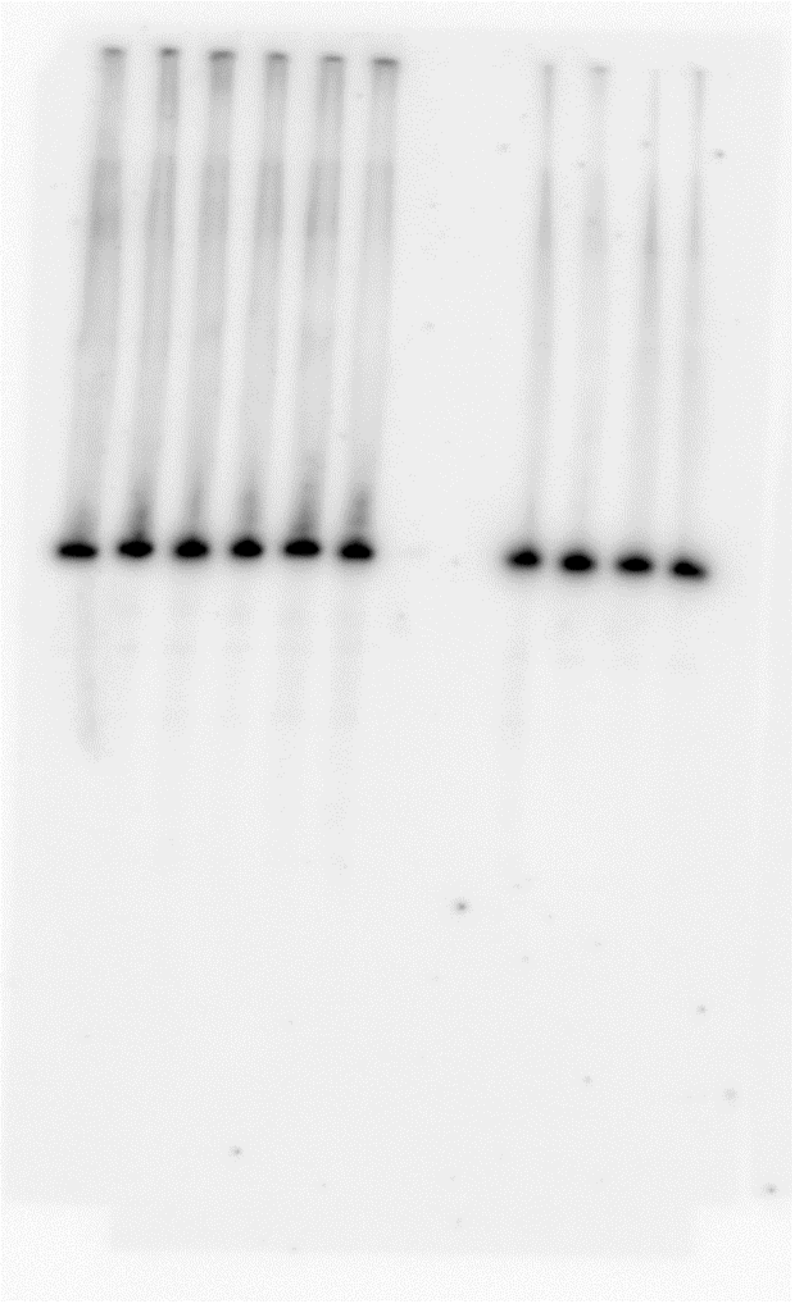

Supplementary Figure 10.

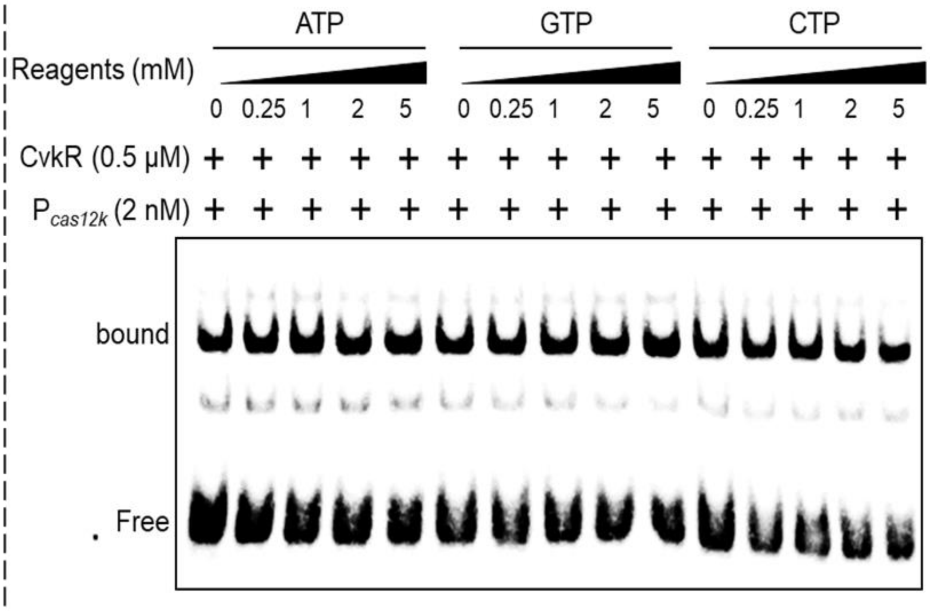

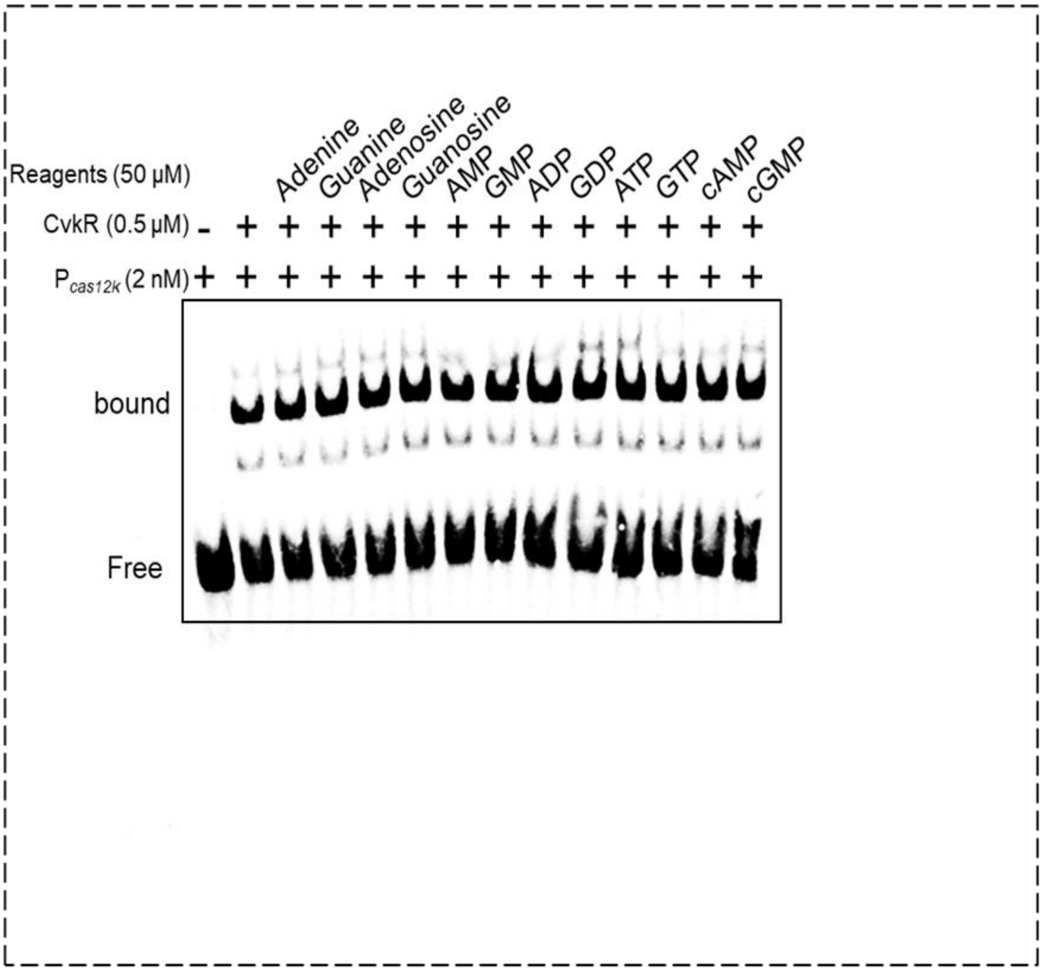

Reagents (50 μM)
Adenine
Guanine
Adenosine
Guanosine
AMP
GMP
ADP
GDP
ATP
GTP
cAMP
cGMP
CvkR (0.5 μM)
P cas12k (2 nM)
bound
Free

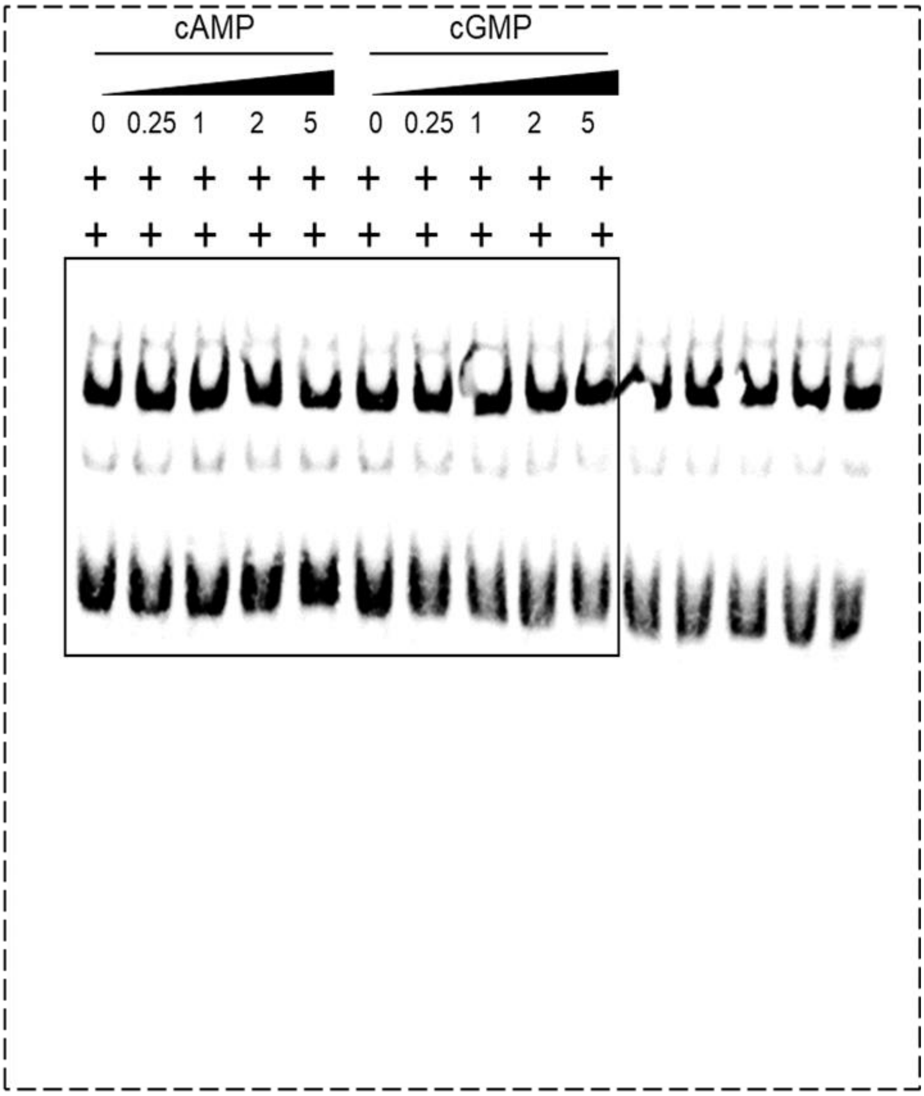

cAMP
cGMP
0 0.25 1 2 5 0 0.25 1 2 5
+ + + + + + + + + +
+ + + + + + + + + +

Supplementary Figure 12.

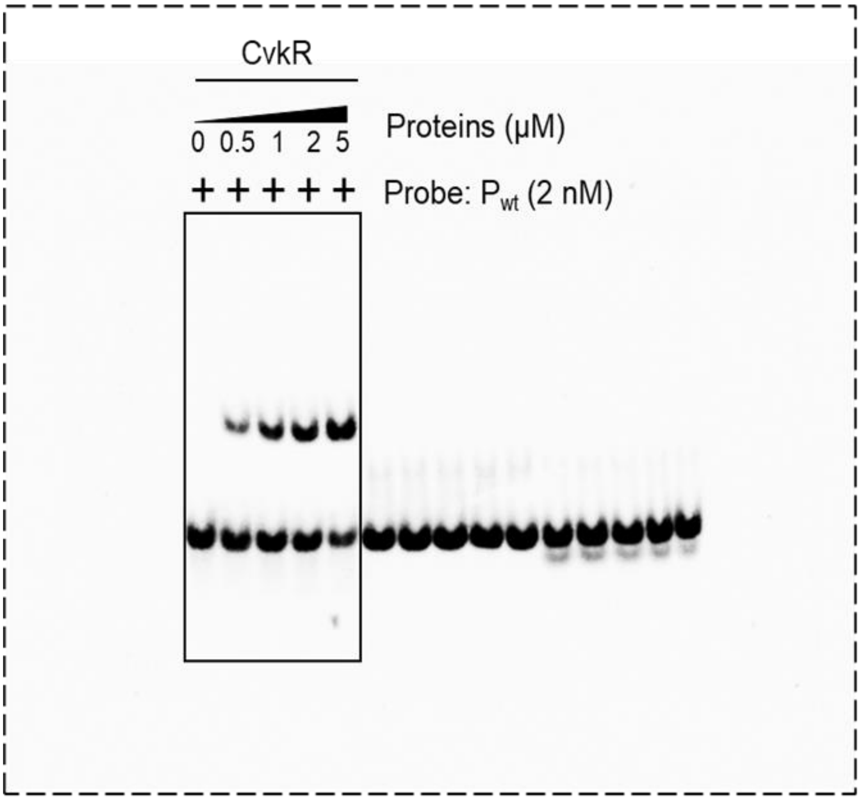

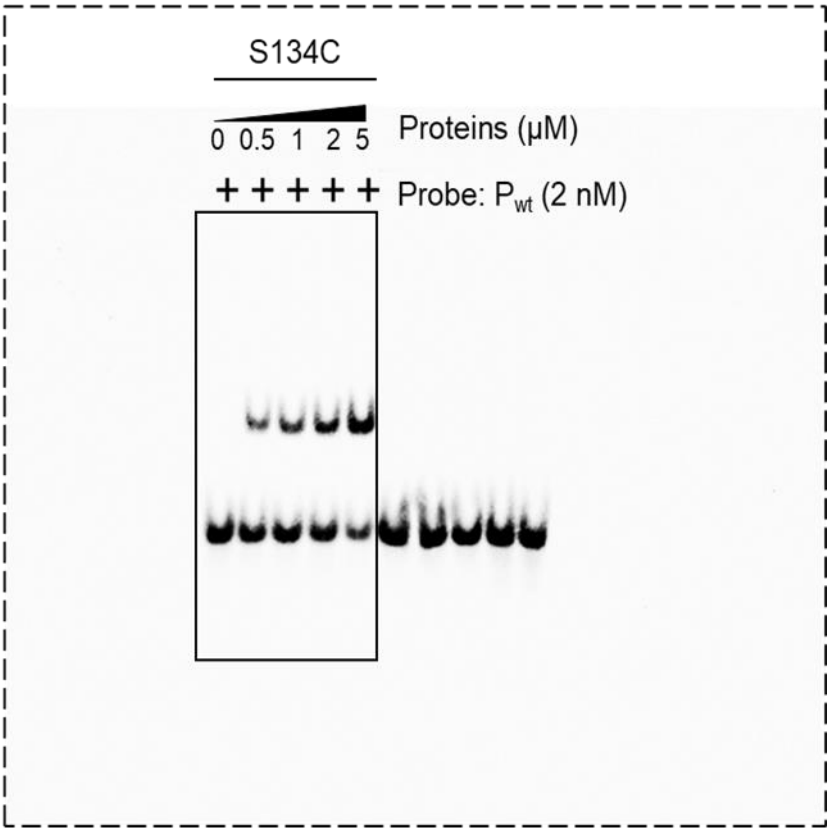

Supplement: Supplementary file 1 — Supplementary Information [file 41467_2023_36542_MOESM1_ESM.pdf]
