# Supplementary material for: CvkR is a MerR-type transcriptional repressor of class 2 type V-K CRISPR-associated transposase systems
Source: Nat Commun. 2023 Feb 18;14:924. doi: 10.1038/s41467-023-36542-9 (PMC9938897; doi:10.1038/s41467-023-36542-9)
Supplement: Supplementary file 3 — Description of Additional Supplementary Files [file 41467_2023_36542_MOESM3_ESM.pdf]

File name: Supplementary Data 1

Description: Details of 118 detected CAST systems in cyanobacteria. Accession numbers and deduced amino acid sequences are given for all identified Cas12k and CvkR homologs (columns A to F), followed by the respective strain names and genome or contig accession numbers (columns G and H). The insertion elements LE always refer to the IS-element closer to the *cas12k* gene and RE refers to the opposite end (columns I and J). The detected *cas12k* and *cvkR* genes were compared to each other and in some cases, we corrected the start codon positions compared to the NCBI annotation, as detailed for *all3613* and *alr3614* in **Fig. 3** of this work. Those instances of proteins with corrected start codon positions are labeled by the [§] suffix in columns A and D and the resulting shorter sequences are given in columns C and F. Some CAST systems showed likely pseudogenized *cas12k* genes indicated by the suffix [#] in column A. If multiple CAST systems exist in one strain, these were numbered consecutively in column G.

File name: Supplementary Data 2

Description: Multiple sequence alignment of Arc-1 repressors within CAST systems.

File name: Supplementary Data 3

Description: Multiple sequence alignment of Arc-2 repressors within CAST systems.

File name: Supplementary Data 4

Description: Multiple sequence alignment of Cas12k homologs in CLUSTAL format. Related to **Fig. 2** and **Supplementary Fig. 2.**

File name: Supplementary Data 5

Description: Multiple sequence alignment of CvkR homologs in CLUSTAL format. Related to **Supplementary Fig. 3**.
