# Supplementary material for: CvkR is a MerR-type transcriptional repressor of class 2 type V-K CRISPR-associated transposase systems
Source: Nat Commun. 2023 Feb 18;14:924. doi: 10.1038/s41467-023-36542-9 (PMC9938897; doi:10.1038/s41467-023-36542-9)
Supplement: Supplementary file 9 — Reporting Summary [file 41467_2023_36542_MOESM9_ESM.pdf]

nature portfolio

Corresponding author(s): Tao Zhu, Xuefeng Lu, Wolfgang R. Hess

Last updated by author(s): 19/1/23

Reporting Summary

Nature Portfolio wishes to improve the reproducibility of the work that we publish. This form provides structure for consistency and transparency in reporting. For further information on Nature Portfolio policies, see our Editorial Policies and the Editorial Policy Checklist.

Statistics

For all statistical analyses, confirm that the following items are present in the figure legend, table legend, main text, or Methods section.

n/a	Confirmed	
☐	☒	The exact sample size (*n*) for each experimental group/condition, given as a discrete number and unit of measurement
☐	☒	A statement on whether measurements were taken from distinct samples or whether the same sample was measured repeatedly
☐	☒	The statistical test(s) used AND whether they are one- or two-sided<br>*Only common tests should be described solely by name; describe more complex techniques in the Methods section.*
☐	☒	A description of all covariates tested
☐	☒	A description of any assumptions or corrections, such as tests of normality and adjustment for multiple comparisons
☐	☒	A full description of the statistical parameters including central tendency (e.g. means) or other basic estimates (e.g. regression coefficient) AND variation (e.g. standard deviation) or associated estimates of uncertainty (e.g. confidence intervals)
☐	☒	For null hypothesis testing, the test statistic (e.g. *F*, *t*, *r*) with confidence intervals, effect sizes, degrees of freedom and *P* value noted<br>*Give P values as exact values whenever suitable.*
☐	☒	For Bayesian analysis, information on the choice of priors and Markov chain Monte Carlo settings
☒	☐	For hierarchical and complex designs, identification of the appropriate level for tests and full reporting of outcomes
☒	☐	Estimates of effect sizes (e.g. Cohen's *d*, Pearson's *r*), indicating how they were calculated

Our web collection on statistics for biologists contains articles on many of the points above.

Software and code

Policy information about availability of computer code

Data collection	Microarray raw data were obtained in an Agilent microarray Scanner C, Modell G2505C using Agilent Scan Control and Feature Extraction software version 10.7.3.1.<br>The diffraction data were collected at the Shanghai Synchrotron Radiation Facility (SSRF), beamlines BL17U1 and BL18U1, in a 100K nitrogen stream.<br>Dynamic light scattering analyses were performed using a DynaPro99 DLS plate reader instrument (Wyatt) equipped with an 830-nm laser source.<br>In TXTL analyses, fluorescence was measured in a Wallac 1420 Victor2 microplate reader.
Data analysis	Microarray data were processed with the limma R package version 3.52.4 as part of Bioconductor version 3.16.<br>The Bayesian analysis of phylogeny were performed with a strict clock model and a birth-death speciation process (Yule) as tree prior, using the standard parameters (clock.rate: fixed value = 1; treeModel.rootHeight: Using Tree Prior [0, infinity]; and yule.birthRate: LogNormal [1, 1.5], initial = 2) as well as the substitution model Blosum62. The distribution was Markov chain Monte Carlo (MCMC) sampled every 1000 steps over 1 million generations.<br>The diffraction data indexing, integration, and scaling were conducted using software HKL3000, version 721.3. Structure refinements were iteratively performed using the programs Phenix (version 1.17.1-3660) and Coot (version 0.9.8.1 EL). Structural alignments were performed using PyMOL (version 2.4.0).<br>Wyatt Dynamics software (version 7.0) was used to calculate the hydrodynamic radius in the dynamic light scattering analyses.<br>Microsoft Excel 2019 software was used for statistical evaluation.

For manuscripts utilizing custom algorithms or software that are central to the research but not yet described in published literature, software must be made available to editors and reviewers. We strongly encourage code deposition in a community repository (e.g. GitHub). See the Nature Portfolio guidelines for submitting code & software for further information.

Data

Policy information about availability of data

All manuscripts must include a data availability statement. This statement should provide the following information, where applicable:

- Accession codes, unique identifiers, or web links for publicly available datasets
- A description of any restrictions on data availability
- For clinical datasets or third party data, please ensure that the statement adheres to our policy

Source data are provided as a Source Data file. The full transcriptome datasets for the WT and mutants cvkR and cvkRCom are accessible from the GEO database with the accession number GSE183629 [https://www.ncbi.nlm.nih.gov/geo/query/acc.cgi?acc=GSE183629] (Deletion and complementation of alr3614 in Anabaena 7120). The structural data can be accessed at the Protein Data Bank under the PDB accession number 7XN2 [http://doi.org/10.2210/pdb7XN2/pdb]. Other PDB structures referred to in this study are 1Q07 [http://doi.org/10.2210/pdb1Q07/pdb] (CueR structure), 4R4E [http://doi.org/10.2210/pdb4R4E/pdb] (GlnR-DNA structure), and 5D8C [http://doi.org/10.2210/pdb5D8C/pdb] (HiNmlR-DNA structure). AlphaFold2 predicted McdR (NCBI: ABK74795.1) [https://alphafold.ebi.ac.uk/entry/A0QYF9].

Human research participants

Policy information about studies involving human research participants and Sex and Gender in Research.

Reporting on sex and gender	N/A
Population characteristics	N/A
Recruitment	N/A
Ethics oversight	N/A

Note that full information on the approval of the study protocol must also be provided in the manuscript.

Field-specific reporting

Please select the one below that is the best fit for your research. If you are not sure, read the appropriate sections before making your selection.

☒ Life sciences ☐ Behavioural & social sciences ☐ Ecological, evolutionary & environmental sciences

For a reference copy of the document with all sections, see nature.com/documents/nr-reporting-summary-flat.pdf

Life sciences study design

All studies must disclose on these points even when the disclosure is negative.

Sample size	The current work doesn't involve the analysis of populations. For functional assays, the number of times that experiments were repeated is addressed under "Replication" below.
Data exclusions	No data was excluded.
Replication	All biochemical and in vitro assays were repeated several times independently with at least duplicate samples per assay. The detailed replicates for each experiments are given in the manuscript. All attempts at replication were successful.
Randomization	Randomization was not relevant because our study did not involve the allocation of samples/organisms/participants into experimental groups.
Blinding	Investigators were not blinded to group allocation because group allocation was not involved in our study. Investigators were not blinded during data collection because the collected data were quantitative in nature (such as bands in gel blot analyses or fluorescence values) and were not prone to subjective interpretation.

Reporting for specific materials, systems and methods

We require information from authors about some types of materials, experimental systems and methods used in many studies. Here, indicate whether each material, system or method listed is relevant to your study. If you are not sure if a list item applies to your research, read the appropriate section before selecting a response.

Materials & experimental systems

n/a	Involved in the study
☐	☒ Antibodies
☒	☐ Eukaryotic cell lines
☒	☐ Palaeontology and archaeology
☒	☐ Animals and other organisms
☒	☐ Clinical data
☒	☐ Dual use research of concern

Methods

n/a	Involved in the study
☒	☐ ChIP-seq
☒	☐ Flow cytometry
☒	☐ MRI-based neuroimaging

Antibodies

Antibodies used

Anti-DYKDDDDK (FLAG tag) Mouse Monoclonal Antibody (TransGen); Goat Anti-Mouse IgG/AP (Solarbio)

Validation

Anti-DYKDDDDK (FLAG tag) Mouse Monoclonal Antibody (#HT201, 1:3000, TransGen) was validated by the manufacturer by western blot against FLAG-tag fusion protein in cell lysate (https://www.transgen.com/antibody_tag/371.html).
Goat Anti-Mouse IgG/AP (#K0031G, 1:3000, Solarbio) was validated by the manufacturer by western blot against mouse primary antibodies that target specific proteins from cell lysates (https://www.solarbio.com/goods.php?id=66151).
